# Determining half-life of SARS-CoV-2 antigen in respiratory secretion

**DOI:** 10.1007/s11356-023-27326-1

**Published:** 2023-05-02

**Authors:** Yang Guang, Liu Hui

**Affiliations:** Department of Laboratory and Quarantine, Dalian Medical University, Dalian, 116044 China

**Keywords:** SARS-CoV-2, COVID-19, Half-life, Environmental hygiene

## Abstract

Severe acute respiratory syndrome coronavirus 2 (SARS-CoV-2) is primarily transmitted from person to person through respiratory droplets and aerosols. It is also possible for the virus to be transmitted indirectly through environmental contamination. The likelihood of environmental transmission depends on several factors, including the survival time of the virus in respiratory secretions. However, the stability of SARS-CoV-2 in respiratory secretions has not been investigated. In this study, we compared the half-life of the SARS-CoV-2 antigen in respiratory secretion under different conditions. We applied respiratory secretion (5 µL) to glass slides, air-dried the slides for 1 h, and kept them at 24 °C or 4 °C for 10 days. Respiratory secretions were also placed in test tubes (sealed to preserve moisture) and in normal saline for 10 days. The concentration of SARS-CoV-2 antigen in all samples was simultaneously measured using colloidal gold immunochromatography, and the half-life of the antigen was calculated. The half-life of the antigen in the wet (sealed tube) and saline samples at room temperature was 5.0 and 2.92 days, respectively. The half-life of the antigen in the air-dried sample at room temperature and at 4 °C was 2.93 and 11.4 days, respectively. The half-life was longer in respiratory secretions than that in normal saline. The half-life was also longer in respiratory secretions, at a lower temperature, and under wet conditions. Therefore, environmental transmission can also play a significant role in the spread of the virus. Robust prevention and control strategies could be developed based on the half-life of the antigen in respiratory secretions.

## Introduction

Coronaviruses are a group of ubiquitous RNA viruses that are made up of a capsid and a linear single-stranded positive-stranded genome (Shereen et al. 2020; Weiss and Leibowitz 2011; Assiri et al. 2013). Some coronaviruses infect humans and cause diseases, such as Middle East respiratory syndrome (MERS), severe acute respiratory syndrome (SARS), and severe acute respiratory syndrome coronavirus 2 (SARS-CoV-2) (de Groot et al. 2013; Drosten et al. 2003; Rota et al. 2003). The coronavirus disease 2019 (COVID-19) caused by SARS-CoV-2 has become a global public health problem since its outbreak (Maqbool et al. 2023; Augustynowicz et al. 2022; Fengjiao et al. 2022).

The main clinical manifestations of COVID-19 are fever, malaise, and dry cough; SARS-CoV-2 is mainly present in the respiratory secretions of COVID-19-infected patients, who are the main source of infection (Panahi et al. 2023; Li et al. 2020; Jiang et al. 2021). The infection is transmitted via the following two modes: direct transmission, which includes respiratory droplet transmission when the infected person speaks, coughs, and sneezes, and aerosol transmission; indirect transmission, also known as environmental transmission, which includes that virus-carrying droplets or aerosols contaminate the environment and are subsequently picked up by another person.

Environmental transmission can occur when an infected person touches their mouth or nose and then touches a surface, leaving behind virus-carrying droplets. Another person can then touch the contaminated surface and inadvertently transfer the virus to their mouth, nose, or eyes (Krishan and Kanchan 2020; Sharun et al. 2021; Marquès and Domingo 2021).

Environmental transmission can also occur through the inhalation of virus-carrying droplets or aerosols in the environment rather than that in close contact situations, such as when people are talking, coughing, or sneezing near each other, leading to direct transmission. This can happen in poorly ventilated spaces where the virus can accumulate and remain suspended in the air for longer periods (Coccia. 2023a).

Environmental transmission is particularly concerning for SARS-CoV-2 that have a long survival time in respiratory secretions. However, detailed transmission mechanisms and potential transmission routes have not been elucidated. Therefore, understanding the mode of survival of SARS-CoV-2 in respiratory secretions and the stability of the pathogen under different conditions is important for risk assessment and control of SARS-CoV-2 transmission.

Most previous studies have investigated the survival time of SARS-CoV-2 under different conditions using isolated strains (Onianwa et al. 2022; Geng and Wang. 2023; Hirose et al. 2022). The main drawback of these studies is that the stability of the virus was not investigated in its natural habitat, i.e., respiratory secretions; therefore, the effect of respiratory secretion on the virus was not considered. Respiratory secretions contain several biologically active substances that either inhibit or protect against viruses, making it necessary to investigate the effect of respiratory secretions on viruses. However, data on the survival time of SARS-CoV-2 in respiratory secretions are limited.

Environmental transmission may play a significant role in the spread of SARS-CoV-2, particularly if the virus has a long survival time in respiratory secretions. It may be important to maintain good hygiene practices, wear face masks, and improve ventilation and air filtration to reduce the risk of transmission (Benati and Coccia. 2022; Coccia. 2023b, 2021). In this study, we investigated the stability of SARS-CoV-2 by comparing the half-life of the virus (antigen) in respiratory secretions under different conditions. The results of this study will provide a new basis for determining the survival time and mechanisms of SARS-CoV-2 in respiratory secretions and its mode of transmission.

## Materials and methods

### Sample and data

**Fig. 1:**
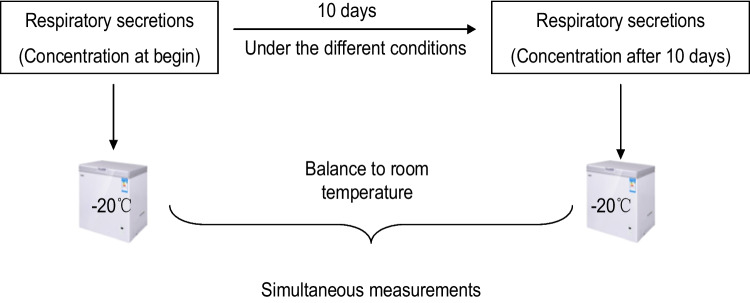
Workflow of the study

A respiratory secretion sample was obtained from a patient infected with SARS-CoV-2 (the author) in the catarrhal phase, and the sample tested positive for SARS-CoV-2 antigen. Figure 1 presents the process followed to determine antigen concentration in respiratory secretion samples on days 0 and 10 of storage simultaneously. The samples from day 0 and the treated samples obtained after incubation at different temperatures on day 10 were stored in a refrigerator at − 20 °C, and all specimens were equilibrated at room temperature for further analysis.

### Measures of variables

The antigen assay was performed using a colloidal gold immunochromatography-based SARS-CoV-2 antigen rapid test kit (Tianjin Bioscience Company, China) as per the manufacturer’s instructions. To ensure that an accurate amount of the sample was added to the test device, a pipette (100 µL) was used. The color was allowed to develop for 15–20 min, and images of the device were taken. The color bands in the photo were quantified using the Image J scanning software. On the device, C was the quality control band and T was the test band. The grayscale of C and T bands was obtained, and ratio (ratio = T/C) was defined as the relative concentration of the antigen.

For the standard curve preparation, a total of 5 µL of respiratory secretion sample was added to the buffer (300 µL) provided in the test kit and mixed thoroughly as the original tube; five tubes with 1:1 (original), 1:2, 1:4, 1:8, and 1:16 dilutions were prepared and were recorded as 1:1, 1:2, 1:4, 1:8, and 1:16, respectively. The diluted samples were tested using the antigen test kit; the obtained results were quantitatively assessed after 15–20 min and ratio-value was calculated.

For the determination of the half-life of the antigen under dry conditions, five microliters of the respiratory secretion sample was smeared on glass slides, and the slides were air-dried for 1 h. Next, the slides were placed in the refrigerator (4 °C) or at room temperature (24 °C) (Fig. 1) for 10 days; antigen concentration was tested simultaneously for all samples after the slides were equilibrated at room temperature. A swab stick dipped in the buffer provided in the kit was used to collect the sample from the slides. The swab stick was repeatedly stirred in the buffer for 30 s, and antigen concentration was measured.

For antigens in different conditions (moist and saline), the secretion group included two test tubes with respiratory secretion samples that were sealed to preserve moisture, and one tube was placed at − 20 °C and the other at room temperature for 10 days; then, the antigen concentration in each sample was simultaneously measured.

For saline treatment, 5 µL of respiratory secretion sample was added to 50 µL of saline, and the samples were incubated at the following two temperatures: − 20 °C and room temperature. After 10 days, 50 µL of the liquid in the tube was added to the sample buffer (300 µL) provided in the antigen kit and mixed thoroughly, and the antigen concentration was measured.

### Model and data analysis procedure

For the standard curve preparation, the antigen concentration in the initial sample was considered as 1000 U, and the expected antigen concentrations of the antigens in the remaining four tubes were considered to be 500, 250, 125, and 62.5 U, respectively. The expected antigen concentration was converted to its logarithm value (LnU), and the relationship between LnU and ratio-value was determined, and a regression equation was established. Statistical analyses used SPSS software (SPSS, Chicago, IL, USA), and a value of *P* < 0.05 (bilateral) was considered statistically significant.

For half-life calculation, the antigen concentration (U-value) was calculated from the ratio-value according to the regression equation, where C_0_ was the initial antigen concentration, and C_10_ was the antigen concentration after 10 days (*t* = 10). The half-life (T_1/2_) of the antigen was calculated using the following equation (Wang and Liu 2022):

1 $${\mathrm{T}}_{1/2}=0.963/\mathrm{k};\;\mathrm{k}=\left({\mathrm{LnC}}_{\mathrm{0}}-{\mathrm{LnC}}_{\mathrm{10}}\right)/\mathrm{t}$$

## Results

The results of the antigen assay were performed using serially diluted samples. Data was shown in Table 1. The ratio-value was linearly related to the LnU of the sample (*r* = 0.987, *P* = 0.002). The regression equation developed based on LnU is as follows:

**Table 1:** Original data and the relationship between ratio-value and LnU

Unit	LnU	Gray scale		Ratio (T/C)	Coefficient	*P*-value
		C	T			
1000.000	6.908	131.198	117.546	0.896		
500.000	6.215	135.127	106.898	0.791	Constant: − 2.462	0.047
250.000	5.521	139.940	103.337	0.738	Ratio: 10.663	0.002
125.000	4.828	153.306	105.159	0.686	Adjusted R^2^: 0.965	0.002
62.500	4.135	158.467	100.137	0.632		

2 $$\mathrm{LnU}=10.663\mathrm{Ratio}-2.462$$

And U-value was calculated using the following equation:

3 $$\mathrm{U}={\mathrm{e}}^{\left(10.663\mathrm{Ratio}-2.462\right)}$$

Antigen concentrations of the air-dried samples at room temperature and at 4 °C after 10 days were calculated according to Eqs. (2) and (3) as shown in Table 2; antigen concentration of the samples in sealed test tubes at room temperature after 10 days and that of the saline samples is also shown in Table 2. The half-life of the antigen under different conditions was obtained according to Eq. (1) and is presented in Table 2. The half-life of the antigen in the air-dried samples was 2.93 and 11.4 days at room temperature and 4 °C, respectively. The half-life in the moist and saline samples at room temperature was 5.0 and 2.92 days, respectively.

**Table 2:** Half-life of SARS-CoV-2 antigen in respiratory secretion samples under different conditions

Groups	Temperature (°C)	Ratio-value		Concentration (U)		T_1/2_ (d)
		R_0_	R_10_	C_0_	C_10_	
Air-dried (smear)	24	0.88	0.66	1013.00	95.37	2.93
Air-dried (smear)	4	0.88	0.82	1013.00	551.24	11.39
Moist (tube method)	24	0.69	0.56	134.50	33.61	5.00
Normal saline	24	0.86	0.64	863.36	80.62	2.92

## Discussion

Normally, SARS-CoV-2 is present in respiratory secretions, and the secretions evaporate rapidly in the environment. Therefore, in this study, we simulated natural environmental conditions for the virus and prepared secretion smears to determine the half-life of the virus. Viruses in respiratory secretions are commonly detected using colloidal gold immunochromatography; therefore, this technique was used in this study. The present study demonstrated that quantitative injection and strip scanning techniques could be used to quantify relevant antigens, which laid the foundation for the subsequent study.

Our results showed that the half-life of the antigen in the air-dried samples was 2.93 and 11.4 days at room temperature and 4 °C, respectively. The half-life in the moist and saline samples at room temperature was 5.0 and 2.92 days, respectively.

The half-life in the normal saline sample at room temperature was 2.92 days, which was close to the half-life values obtained using virus culture-based experiments (1.7–2.7 days) (Riddell et al. 2020). It is suggested that the half-life values obtained using the measuring antigen method are reasonable. Moreover, the method of measuring antigens should have better methodological reliability than virus culture-based experiments.

Interestingly, the virus had a shorter half-life in saline than that in moist respiratory secretions, which could be because the molecules present in respiratory secretions protect the virus, suggesting that the viability of the virus should not be the same within and without respiratory secretions. The assessment of the half-life of a viral antigen usually adopts isolated strains and virus cultures (Riddell et al. 2020; Onianwa et al. 2022; Hirose et al. 2022). Our findings suggest that assessment of the half-life using isolated strains or virus cultures may be inaccurate because isolated strains or virus cultures are not provided their natural habitat (respiratory secretions). Methods of isolated strains and virus cultures could underestimate the persistence of SARS-CoV-2 in their natural habitat.

The results also revealed that the half-life at 4 °C was remarkably more than that at room temperature, suggesting that the virus could survive longer in winter or cold chain logistics. At room temperature, the virus had a longer half-life under moist conditions than under dry conditions, suggesting that virus survival is better under moist conditions. The half-life values obtained using virus culture-based experiments are mostly in the range of several hours to a few days, which are lesser than the results obtained in this study (Ijaz et al. 2021; Matson et al. 2020; Harbourt et al. 2020). We believe that virus culture–based studies use sensitive cell lines. Nevertheless, the sensitivity of these cells to viruses may be lower than that of humans; therefore, cell culture data cannot be extrapolated and applied to developing treatment and prevention strategies for humans.

The antigen-based half-life assay is a test based on the destruction of the viral surface, which is different from cell culture and viral nucleic acid assays; therefore, direct detection of SARS-CoV-2 antigen may more appropriately reflect infectivity compared to RNA detection (Wang et al. 2003a; Peck Palmer et al. 2023; Hillig et al. 2023; Wang et al. 2023b); our results could be more reliable. Moreover, the half-life of the RNA determined by PCR could be more than the actual duration of infectivity.

## Conclusions

In summary, the half-life of the SARS-CoV-2 antigen in respiratory secretion was compared under different conditions. The results revealed that the half-life was longer in respiratory secretions and also longer under wet conditions and at a lower temperature. One limitation of this study is that the sensitivity of colloidal gold immunochromatography could slightly be lower than that of RNA detection. However, a moderate conservative estimate can be obtained by our method. Present studies have shown that SARS-CoV-2 can survive for up to several days or more in respiratory secretions. This highlights the importance of maintaining good hygiene practices, such as regular hand washing, proper ventilation, and air filtration, which can help to reduce the risk of environmental transmission. Overall, while direct transmission is the primary mode of transmission for SARS-CoV-2, environmental transmission can also play a significant role in the spread of the virus. The use of antigens, especially the half-life of antigens in respiratory secretions, as a reference for risk assessment of objects contaminated with respiratory secretions may be more effective and reliable for the development of robust prevention and control strategies.

## Data Availability

All relevant data are within the paper and no additional data are available.
